# Modeling the COVID-19 spread, a case study of Egypt

**DOI:** 10.1186/s42787-021-00122-x

**Published:** 2021-05-21

**Authors:** Assem S. Deif, Sahar A. El-Naggar

**Affiliations:** 1 Engineering Mathematics and Physics Department, Faculty of Engineering, Cairo University, Giza, 12613 Egypt; 2 Misr University for Science and Technology, Cairo, Egypt

**Keywords:** COVID-19, Mathematical model, Epidemiology, 92-10, 92D30

## Abstract

In this article, the authors applied a logistic growth model explaining the dynamics of the spread of COVID-19 in Egypt. The model which is simple follows well-known premises in population dynamics. Our aim is to calculate an approximate estimate of the total number of infected persons during the course of the disease. The model predicted—to a high degree of correctness—the timing of the *pandemic peak* $t_{{\text{m}}}$ and the *final epidemic size* $P$; the latter was foreseen by the model long before it was announced by the Egyptian authorities. The estimated values from the model were also found to match significantly with the nation reported data during the course of the disease. The period in which we applied the model was from the first of April 2020 until the beginning of October of the same year. By the time the manuscript was returned for revision, the second wave swept through Egypt and the authors felt obliged to renew their study. Finally, a comparison is made with the SIR model showing that ours is much simpler; yet leading to the same results.

## Introduction

Models for predicting how epidemics are spread in a certain community are not only numerous but also dating from the early twentieth century. They are of two kinds: the first is statistical in nature; i.e. like fitting data to a curve and then extrapolating from there. The second are the mechanistic models simulating future transmission scenarios under a list of assumptions. The first type is usually used for short-term forecasts using machine learning or regression allowing for quick projections that are useful for policy makers. The famous model presented by the Institute for Health Metrics and Evaluation (IHME) falls into this category [1]. These models are generally not suited for long-term predictions; to the least anticipating the date of the pandemic peak. On the contrary, mechanistic models are well suited for epidemiological dynamics like when the peak will occur or whether a revival will happen. They can also deal better with large populations. They also allow for nonlinear feedback; like the more persons are infected the faster the pandemic spreads. Therefore, they are well-fitted for inference about intervention efficacy. The model we applied is a logistic growth model which falls into this category.

Of the early mechanistic models is the SIR; short for Susceptible–Infected–Recovered, which was designed by Kermack and McKendrick [2]. In this model, the authors considered a fixed population by modeling three variables: the susceptible, the infected and the recovered. They are called compartments because people may progress between them. The order of the label denotes the flow pattern from left to right. Like it is understood, the first group is not yet infected; yet it can be at any time, the second group comprises those already infected and can therefore transmit the disease to others, while the last group although at one time contracted the disease but are now recovered. The model goes on describing the rules upon which the flow proceeds between these three categories. In fact, the SIR model paved the way for future research with some alterations or say assumptions; e.g. the SEIR model (Susceptible–Exposed–Infectious–Recovered) or SEIRS (susceptible–exposed–infectious–recovered–susceptible) types, depending on whether the acquired immunity is permanent or not. In other words, a distinction is made between those models describing diseases that grant lifelong immunity and those which do not. The first case leads to the so-called SIR type models, the second to the SIS type models. Some models further added extensive complexities like a nonlinear transmission rate leading to some chaotic and hyper-chaotic dynamical behaviors [3]. Some investigated the uncertainty of the parameters fed into the model [4, 5]; e.g. health care management, the natural history of the disease, possible survey biases, the transmission behavioral factors, the sampling or measurement errors, etc.… Determining the sensitivities to changes in these parameter values will help identify those key parameters that can help the decision makers explore various policies [6]. In general, not that modeling needs a laborious effort on the part of the modeler, but demands also a substantial team of specialists, whether in virology, medicine, epidemiology, ecology, public health, policy makers, statistics, computer simulation experts, etc.… and sponsored by large institutions [7]. That is due to the many assumptions and parameters involved in the study. The latter usually lasts several months in order to test conceptual results such as thresholds, the transmission characteristics of the disease, how can the basic reproduction number be monitored regularly, assessing quantitative conjectures, whether the model applies with/without therapy intervention, what/how crucial data are collected. Again, whether the incubation period varies from one person to another, what is the average recovery period, how and when antibodies develop and whether the person remains contagious for a while, etc.… A simple review to the literature reveals the rapid growth of epidemiology modeling by researchers and how diverse is the list of acronyms in describing the flow patterns between the different compartments. A few examples only of those acronyms are the SIR, SIS, SEI, SEIS, SIRS, SEIR, SEIRS models and so on….

Models also make different assumptions about the properties of each specific pandemic. For the novel corona-virus, how infectious it is, or what is the rate at which people die as a result of acquiring the infection. Again, the way the disease is transferred from one person to another which varies according to preventive measures and social distancing, the probability of being infectious, what if a proportion of the population is immunized at birth by vaccination, etc.… All these are incorporated in some dimensionless parameter $R_{{\text{o}}}$ called the *basic reproductive number*. It represents the average number of people that a single infectious person will infect over the course of his/her infection; a parameter which takes an arduous effort for its calculation [8]. If $\beta$ represents the average infection-producing contacts per unit time, with a *mean infectious period* of $1/\gamma$, then $R_{{\text{o}}} = \beta /\gamma$. For example COVID-19 (Corona Virus Disease 2019) has an $R_{o}$ of approximately 4, so, on average a person who has COVID-19 will pass it on to 4 other people.

The proposed model will be used to forecast the growth of the *number of cumulative cases* $N(t)$ of infected persons in Egypt; being our case-study. In other words, we target the total number of persons—during the whole course of the disease—who are already infected on a daily basis in addition to those recovered or died since they contracted the disease at some previous time. We also used the model to predict both the timing of the peak $t_{{\text{m}}}$ as well as the final epidemic size *P*. For our model to be sound, $N(t)$ must match the data advertised by the WHO (World Health Organization) site [9] appearing in the first column of its table under the heading of total cases. So a number like 3,617,408 published on 7–15-2020 in the first column for the USA denotes the cumulative total number of individuals who contracted the disease whether now or before; i.e. during its whole course so far*.*

To isolate the set of infected persons $I$ from the recovered set $R$ is unimportant in our view; while one group is at present contracting the disease; the other did already contract it at some previous instant of time; so it is only a matter of time span between both groups. This explains why the total number of infected cases *N* announced in the first column in [9] carrying the sum of both figures is the dominant data which any country declares. Besides, not only that the recovered persons are a proper subset of *N*, but it will also become the most obvious source of uncertainty affecting not only this model but all models in that we don't know how many persons are, or have been infected. In our view, the ratio of the recovered to the infectives is relevant only to the policy makers so that they are informed prematurely of the recovery rate they can expect independent of any medical intervention. But sure this ratio is not something that the model can anticipate beforehand. Any negligence on the part of the healthcare system or the unintentional absence of any safeguarding measures to face unexpected shortcomings which may arise can lead to far-reaching consequences. For example, a very common instance occurring is the sudden lack of oxygen in hospitals at a crucial moment amounting to high mortality; and surely no model can take it into consideration as a possible risk factor. Thus the less representative variables in the model, the less will be their deviation from the true data. Again, most models assume unrealistically that the population is uniform, i.e. constituting a homogeneous mixture which is not true. It is commonly known that children usually have more adequate contacts per day than adults. Again, different geographic groups and socio-economic ones usually have different contact rates. Besides, who can tell if any child belongs to the class M having temporary passive immunity to infection acquired from his mother during pregnancy; whether he already moved to the susceptible class or not yet, etc.…But we admit that those issues are difficult to investigate.

## Method

It is agreed upon that pandemics spread according to the exponential function; a function investigated by Jacob Bernoulli (1655–1705). Let us assume that, at the beginning of the outbreak, the number of infected persons contagious to others is $N_{0}$ which can be one person only (*patient zero*). This will result in a number $N_{{\text{o}}} \times R_{{\text{o}}}$ of the newly infected persons. Thus, the total number of infected persons per one cycle of contagion becomes equal to $N_{{\text{o}}} + N_{{\text{o}}} \times R_{{\text{o}}} = N_{{\text{o}}} (1 + R_{{\text{o}}} )$. As the number of cycles *n* unfolds each of period $1/\gamma$, the bracket $(1 + R_{{\text{o}}} )$ is raised to the power $n = t\gamma$ after a time $t$. Thus, the total cumulative number $N$ of infected cases at time $t$ becomes equal to $N(t) = N_{o} (1 + R_{o} )^{t\gamma } = N_{o} e^{{t\gamma \ln (1 + R_{o} )}}$, where "ln" stands for the natural logarithm. But $\ln (1 + R_{o} ) < R_{o}$; thus the rate ${\text{d}}N/{\text{d}}t$ with which the cumulative number $N$ of infected cases grows at time $t$ is bounded by $N \times \gamma \times R_{o}$; and the model is therefore governed by the simple differential equation ${\text{d}}N/{\text{d}}t = N\beta$ having as solution $N = N_{0} e^{\beta t}$.

The reason for accounting for $N_{0}$ as contributing to the next cycle of infection although this group might have recovered is because it usually remains active for a longer period and might continue spreading the disease. For it is quite known that in general, patients may become infectious on average 4 days before showing major symptoms (a susceptible individual first goes through a latent period after infection before becoming infectious) in addition to ten more days at least since symptoms first appeared until recovery. In this long period of illness overlapping the 14 days of incubation period of $N(t) \times R_{o}$, $N(t)$ remains active; and therefore the original $N(t)$ can simultaneously transmit it. So being cautious, we add it to the group which can spread the disease. Besides, there are cases reported by the MIT medical issue May-13–2020, in which the person can still test positive after being symptom free for more than 2 weeks.

On the contrary, if $N_{{\text{o}}}$ is exempted from the responsibility of a secondary infection, the second cycle gives $N_{{\text{o}}} + N_{{\text{o}}} \times R_{{\text{o}}} (1 + R_{{\text{o}}} ) = N_{{\text{o}}} (1 + R_{{\text{o}}} + R_{{\text{o}}}^{2} )$ as the total number of infected persons, and so on…; i.e. a geometric series in $R_{{\text{o}}}$ having as sum

1 $$N(t) = N_{{\text{o}}} \frac{{R_{{\text{o}}}^{t\gamma + 1} - 1}}{{R_{{\text{o}}} - 1}} = N_{{\text{o}}} \frac{{R_{{\text{o}}} {\text{e}}^{{t\gamma \ln R_{{\text{o}}} }} - 1}}{{R_{{\text{o}}} - 1}}.$$

The advantage of this model over the previous one, is that it is commonly known that if $R_{{\text{o}}} < 1$, the spread must eventually die out (*N* becomes constant and ${\text{d}}N/{\text{d}}t \to 0$) which doesn't show in the previous model. For if $R_{{\text{o}}} < 1$, $\ln R_{{\text{o}}} < 0$ and the exponential function in Eq. (1) will vanish as $t \to \infty$, and *N* will stabilize to

2 $$N_{\infty } = \frac{{N_{{\text{o}}} }}{{1 - R_{{\text{o}}} }},\quad \left( {R_{{\text{o}}} < 1} \right)$$

The data though reported by the different countries for the COVID-19 doesn't run according to the above simplified model even if $R_{{\text{o}}}$ is big, otherwise we should have witnessed a much dramatic explosion in the number of infected cases. Thus, the above suggested equation is suitable to represent the dynamics of COVID-19 only in the beginning of the outbreak. For although $R_{{\text{o}}}$ is not small for COVID-19 as given in [10] (exceptional small values have been recorded [11]), the actual data reported worldwide show some saturation; for there are many unsusceptible person, being immune or live in remote areas, etc.…. The authors preferred therefore to rely on the actual universal data published by the WHO [9] from which an average *growth rate* $c$ substituting $\beta$ can be calculated at the beginning of the outbreak from the expression

3 $$c = \left. {\frac{1}{N}\frac{{{\text{d}}N}}{{{\text{d}}t}}} \right|_{N \cong 0}$$

(The average estimated value from the published data in [9] gives *c* = 0.06 days^−1^). However, by using this value in our model, we found that it gives—over a longer period of time—an expected number of infected cases much higher than the actual value. The truth is, whenever the total number of infected persons $N$ in a certain pandemic becomes large enough, only a few infections will be recorded; for the virus won't find enough people to infect since the majority had been already infected and this is the main idea behind the *herd immunity* (see footnote). This occurs especially in countries with a small population size; since an instant in time will quickly come at which there will be no new susceptible persons at all to be infected. This assumption, being quiet reasonable, convinced the authors to take it from there, and try to modify the model accordingly by adjusting this ratio $c$ by scaling it with a proportionality factor that obeys certain rules: It must be dimensionless like $R_{{\text{o}}}$. That it must be less than unity. That it must tend to zero as $t \to \infty$ and that for small *t*, $N$ should grow with a rate $c$. The reason why it must be dimensionless is straightforward for it is a proportionality factor to the rate $c$. That it must be less than unity is also essential so that the spread of the pandemic declines eventually given enough time; otherwise it will explode through the foregoing exponential function. It must also tend to zero as $t \to \infty$ so that the total number of infected cases *N* saturates or that ${\text{d}}N/{\text{d}}t \to 0$ eventually. Finally, at the beginning of the outbreak, it is expected to follow the Bernoulli model as if nothing can stop its spread. For these reasons, the authors settled for the logistic model introduced by Pierre François Verhulst in 1838 modeling the population growth in ecology, an improvement of Malthus [12] famous population model in which such proportionality factor is chosen as $[1 - (N/\overline{P})]$. In fact, many authors [13–15], concurrently to us, had the same idea as to use the same model for COVID-19. The reason is that since $0 \le N < \overline{P}$, one has that $0 < 1 - (N/\overline{P}) \le 1$. This means that, as the number of infected persons $N$ approaches the final population size $\overline{P}$, $[1 - (N/\overline{P})]$ comes nearer to zero; and the cumulative number *N* will remain constant; so that no new daily rate of infections will be recorded. On the contrary, in countries with large population like Egypt, the value of *N* is usually negligible relative to $\overline{P}$. The spread of the disease will thus follow at the beginning of the outbreak an exponential growth with a rate $c$, a situation we already witnessed. It therefore seems reasonable that so long that there exist villages or district areas that are not yet infected, there is no clear evidence that the outbreak will subside very soon. The situation was different in a country say like Italy, because there was a lockdown for a long time, so that $\overline{P}$ doesn't constitute the whole of the population; rather those exposed only and which saturates to a final epidemic size *P*, a number which is by far less than $\overline{P}$. So that when the Italian government felt that *N* was about to approach all those that are expected to catch the disease eventually based on similar world data, then there was no need for a complete caution; and the responsibility lies in the hands of the citizens in avoiding the gatherings as much as possible. To clarify further, let us take the example of the USA a country of population size of 328 millions. If an epidemic hits the country, would the whole population be susceptible for an infection, definitely not. For there are those who do not necessarily contract the disease, or for some reasons they are immune by birth, others live in a remote area. In fact, scientists could not explain why some suffer from severe symptoms which can lead to death, while others exhibit minor symptoms or nothing at all. Some may need intensive care units and respiratory intervention apparatus and can still die, while others do not even know if they were hit by the virus at all. The reason is that the person might have developed antibodies from a previous attack of say SARS-Cov-1 or Mers; two of the severe respiratory diseases which reached Egypt in 2002–2004 and in late 2019 respectively. This can also explain the small number of infections in Egypt. Scientists call this phenomenon *immunity memory* against the whole Covid family. The authors therefore settled for the factor $[1 - (N/P)]$. We shall explain shortly how to estimate the value of *P*.

Solving for $N(t)$ the differential equation

4 $$\frac{{{\text{d}}N}}{{{\text{d}}t}} = c\left( {1 - \frac{N}{P}} \right) \, N,\quad N(0) = N_{0}$$

we obtain

5 $$N(t) = \frac{{N_{0} P{\text{e}}^{ct} }}{{P - N_{0} + N_{0} {\text{e}}^{ct} }},$$

in which we took $N_{0} = 790$ in the first of April. We then traced the relation between the total number of infected persons *N* against *t* (from April till the date of submission of the original manuscript being late July) for values of *P* between 400,000–600,000. The reason for choosing these estimates for *P*, is that we consulted the WHO tables [9] and found that, as the number of infective cases *N* approached stabilization, they counted worldwide between 0.4–0.7% of the total population size (Italy 0.4%, Sweden 0.7% trying to adopt herd immunity). Some extreme cases exist like 1.1% for the USA or 0.02% for South Korea. For Egypt it should range thus between 400,000–600,000 citizens all in all to be infected during the whole course of the pandemic. Upon taking *P* = 400,000, we found the estimated curve resembling a great deal the actual values yet shifted a little to the right. Since we know well that the actual data are rough figures for the matter of guidance to the government, it was not worth the effort to proceed with an optimization scheme i.e. to minimize the difference between both under some norm, usually norm-2 (Least-squares fit) to obtain *c* and *P*. Instead, we kept decreasing the value of *P* until we found that *P* = 105,000 makes the two figures: the estimated one from the model and the announced one by the authorities fit each other almost perfectly. At the same date we submitted our paper in July, three authors from Alexandria-Egypt adopted several regression models [16] and obtained the final epidemic size *P* to be around 166,760. Ours is more close to the actual figure of 104,000 which was reported by the government in October after our first submission.

**Fig. 1:**
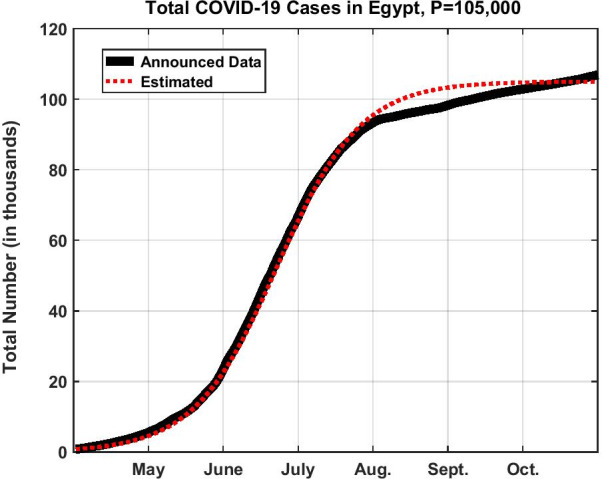
Total number of COVID-19 cases in Egypt in the first wave assuming *P* = 105,000

**Fig. 2:**
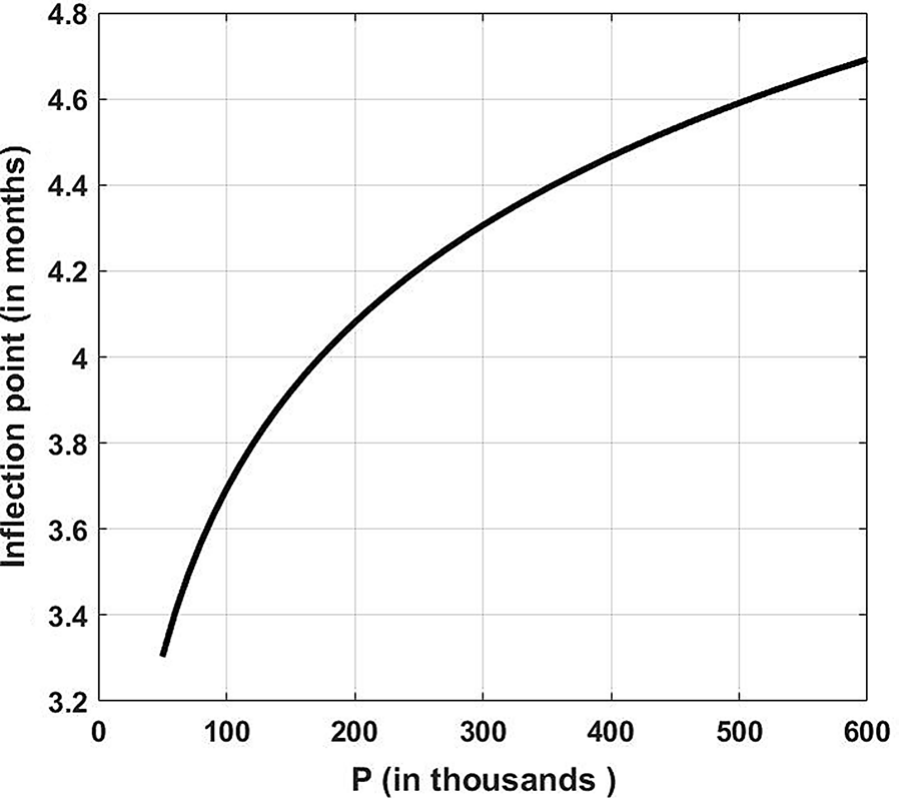
Inflection point in the first wave versus the value of *P*

**Fig. 3:**
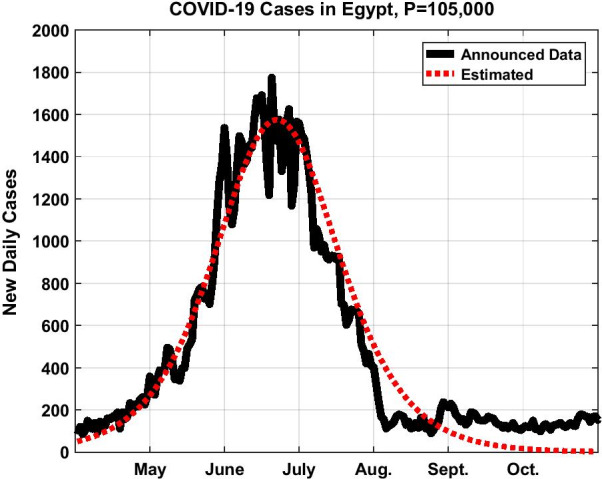
The daily COVID-19 cases in Egypt in the first wave assuming *P* = 105,000

Figure 1 shows $N(t)$ in its *S* shape, while Fig. 2 represents the time remaining to the inflection point as a function of the parameter *P*; all starting from the beginning of April. As we prepared a revised manuscript in December, we preferred to extend the data in Figs. 1 and 3 until the end of October 2020.

From Eq. (5), the graph of $N(t)$ in Fig. 1 saturates to *P*. Such graph is composed of two parts: the first shows that there are always new infections, since

6 $$\frac{{{\text{d}}N}}{{{\text{d}}t}} = \frac{{N_{0} Pc{\text{e}}^{ct} \left( {P - N_{0} } \right)}}{{\left( {P - N_{0} + N_{0} {\text{e}}^{ct} } \right)^{2} }} > 0,\quad {\text{for}}\;{\text{all}}\;t$$

but more important that their number is initially accelerating day after day. This is seen from the second derivative

7 $$\frac{{{\text{d}}^{2} N}}{{{\text{d}}t^{2} }} = N_{0} Pc^{2} {\text{e}}^{ct} \left( {P - N_{0} } \right)\frac{{P - N_{0} - N_{0} {\text{e}}^{ct} }}{{\left( {P - N_{0} + N_{0} {\text{e}}^{ct} } \right)^{3} }},$$

showing that the curve is concave upwards until a point of inflection is reached, a point crucial in the development of the pandemic. To obtain it, we set $P - N_{0} - N_{0} {\text{e}}^{ct} = 0$, giving the timing at which this inflection point occurs; that is

8 $$t_{{\text{m}}} = \frac{1}{c}\ln \frac{{P - N_{0} }}{{N_{0} }} \cong 82\,{\text{days}}$$

(around the 20th of June). Following this date, despite there will be still new infections, but it is the daily rate which starts to lessen day after day (curve becoming concave downwards); meaning that the daily rate reaches its peak value at this point in time. In Eq. (8), $N_{o} < < P$ so that it can be neglected in the numerator relative to *P*. It therefore seems that in general if *c* is large, we reach more quickly the inflection point and the opposite is the case if *c* is small. Equally, if the population *P* is large, it takes a longer time to reach the inflection point. Thus the right procedure to adopt for lessening *P* is to make the people less exposed as much as possible; whether by staying longer times at home or wearing masks regularly. Of course, a lockdown is always possible, except that it leads to bad economic consequences.

Finally, to compare our model with the classical SIR model, ours contains one variable only namely $N(t)$ against three for the SIR. The latter incorporates the following three compartment variables [17]:

9 $$\begin{gathered} {\text{i}})\;\overline{S}(t)\;{\text{the}}\;{\text{number}}\;{\text{of}}\;{\text{susceptible}}\;{\text{people}}\;{\text{at}}\;{\text{time}}\;t \hfill \\ {\text{ii}})\;I(t)\;{\text{the}}\;{\text{number}}\;{\text{of}}\;{\text{infected}}\;{\text{cases}}\;{\text{at}}\;{\text{time}}\;t \hfill \\ {\text{iii}})\;R(t)\;{\text{the}}\;{\text{number}}\;{\text{of}}\;{\text{recovered}}\;{\text{cases}}\;{\text{at}}\;{\text{time}}\;t \hfill \\ \end{gathered}$$

satisfying $\overline{S}(t) + I(t) + R(t) = \overline{P}$ and where $\overline{P}$ is the total population size which is a fixed constant. The equations are

10 $$\begin{gathered} ({\text{a}})\;\frac{{{\text{d}}\overline{S}}}{{{\text{d}}t}} = - \lambda (t)\;\overline{S}(t),\quad \overline{S}(0) = \overline{S}_{0} \hfill \\ ({\text{b}})\;\frac{{{\text{d}}I}}{{{\text{dt}}}} = - \lambda (t)\;\overline{S}(t) - \gamma I(t),\quad I(t) = I_{0} \hfill \\ ({\text{c}})\;\frac{{{\text{d}}R}}{{{\text{d}}t}} = \gamma I(t),\quad R(0) = R_{0} \hfill \\ \end{gathered}$$

All $\overline{S}_{0} \, , \, I_{0 \, } , \, R_{0} \ge 0$ ($R_{0}$ not to be confused with the basic reproduction number).

In our model, $I(t)$ and $R(t)$ are combined instead into one variable $N(t): = I(t) + R(t)$. The function $N(t)$ will thus represent the total cumulative sum found at some instant of time *t* of all those either contracting (infectives) or had previously contracted (recovered from) the disease. Since $\overline{S}(t) = \overline{P} - N(t)$. Equation (10a) will then read

11 $$\frac{{{\text{d (}}\overline{P} - N(t{)}}}{{{\text{d}}t}} = - \frac{{{\text{d }}N(t{)}}}{{{\text{d}}t}} = - \lambda (t)\;\overline{S}(t)$$

giving that

12 $$\frac{{{\text{d }}N(t{)}}}{{{\text{d}}t}} = \lambda (t)\left[ {\overline{P} - N(t)} \right]$$

in which $\lambda (t) \propto \frac{I(t)}{{\overline{P}}}$ or that

13 $$\frac{{{\text{d}}N(t)}}{{{\text{d}}t}} = \, \propto \, \left( {1 - \frac{N(t)}{{\overline{P}}}} \right) \, I(t)$$

Now, look how close is Eqs. (13) to (4); they are identical, except for two things: one, is that the SIR model replaces $N(t)$ in Eq. (4) outside the bracket by $I(t)$. The second, is that $\overline{P}$ in Eq. (13) is constant while $P$ in Eq. (4) is a parameter. For the first distinction, we believe that $N(t)$ was more appropriate to use rather than $I(t)$ in Eq. (13); the fact that at the sudden outbreak of the pandemic $N(t) < < \overline{P}$ so it can be neglected in the equation, and ${\text{d}}N/{\text{d}}t$ must be found proportional to *N* and not *I*. As for the second distinction from the SIR model, it is true that in our case $S(t) + N(t) = P$, but both the variable $S$ and the parameter *P* signify different meanings than those of the SIR model. We already explained that the whole population $\overline{P}$ cannot—for different reasons—be susceptible for an infection, definitely not. For example, the present number announced so far is 85,000 from a total population of 100 millions (that was when we were about to submit our first manuscript). We took into consideration that this constitutes the number of only those that have been tested positive; for there are a vast majority who were not tested or even felt they contracted the disease. But sure this majority could not approach by any means any value near 100 millions and thus we could not take *P* as equal to 100 millions; for it will be totally absurd. Besides, If *P* was to take this value of $\overline{P} = 100,000,000$ like in the SIR model, then by neglecting *N* relative to $\overline{P}$ in Eq. (13), we find that *N* will keep rising continuously without bound; being impossible. On the contrary, from Eq. (5), as $t \to \infty$, $N \to P$ and consequently $S \to 0$. The latter variable *S* will stand only for the number of citizens waiting in the queue to be infected from a totality of 400,000 say for the Egyptian case. So *P* becomes a parameter of choice. In fact, this is one strong point in favor of the model having qualities similar to the statistical models. By taking $P = 105,000$, we found that our estimated data matches exactly the announced ones. This value of 105,000 was never announced by the authorities by the time we submitted the first manuscript, yet our model predicts it as the total expected cumulative number of infectives that can be reached near saturation. From the graph of Fig. 3, it seems that we passed the first peak in early July 2020.

Again, in Eq. (10) adding Eqs. (b) to (c) results in

14 $$\frac{{{\text{d}}I}}{{{\text{dt}}}} + \frac{{{\text{d}}R}}{{{\text{d}}t}} = \frac{{{\text{d}}N}}{{{\text{d}}t}} = - \lambda (t) \, \overline{S}(t)$$

which is again Eq. (11).

The standard SIR model is sometimes criticized for being rigid in its assumptions. For example it assumes a constant basic reproduction number *R*_o_ which is not true. Usually, people respond by lowering their contacts long before herd immunity is attained. The fear to contract the disease influences their behavior and compels them to change their human conduct. Therefore to anticipate massive infections, hospitalizations and deaths in a mechanical way does not appear to be always the case; like in Egypt when a sudden drop in the number of infectives occurred. To say that the model we used is mechanical like the SIR in which the disease grows first exponentially, and then it starts to decline as $N(t)$ approaches *P* is true. But the latter is not fixed like in the SIR; rather a parameter that can be monitored based on the current state of affairs and consequently the rate of growth of $\ln N$ is proportional to $[1 - (N/P)]$. True in both models, whether $\overline{S}(t)$ or $S(t) \to 0$, but in the SIR model $R(t) \to \overline{P}$ (the total population size) which is not possible even under herd immunity. In our model, $N(t) \to P$ (the number of citizens liable for infection); thus we reach the end of the pandemic and the rate of growth becomes equal to zero.

To end our discussion, it is important to mention some new trends in the field. Although the logistic differential Eq. (4) is the classical one to date for modeling the growth of many biological and social science phenomena, yet successful efforts based on fractional calculus emerged lately replacing integer-order models by fractional order ones. It seems that the latter are more descriptive; especially when it comes to hereditary and memory properties of such processes. This trend found applications in numerous disciplines; a famous example is in the growth of tumors in medicine [18]. In [19], the authors replaced the differential operator d/d*t* in Eq. (4) by ${}^{C}D_{t}^{\alpha }$ ($\alpha \in (0,1]$) denoting the fractional differential operator of Caputo type, then used Galerkin method for its solution. While *c* in Eq. (4) still represents the intrinsic growth rate, *P* is called the carrying capacity of the environment. Interestingly enough, the fractional-order predator–prey problem [20] is another variant of Eq. (4) and furnishes an understanding of the dynamics of many biological models. Also, based upon the Khan-Atangana fractional model on the dynamics of the corona virus and which accounts for many compartment variables [21], the authors in [22] proposed a computational method to solve the fractional-order equations along with a stability analysis. In [23] a generalized wavelet method is developed together with the quasi-linearization technique to solve the Volterra's population growth model of fractional order and the problem is finally transformed into an equivalent system of algebraic equations easily solvable using classical methods. Since biological models can exhibit hyperchaotic behaviours, the authors in [24] studied their chaos control in both the frameworks of classical and fractional calculus by designing a set of optimal and adaptive controllers to compensate the undesirable hyperchaotic behaviours. The purpose of this paper is to analyze and control the hyperchaotic behaviours of a biological snap oscillator. They mainly study the chaos control and synchronization of a hyperchaotic model in both the frameworks of classical and fractional calculus, respectively. First, the phase portraits of the considered model and its hyperchaotic attractors are analyzed. Then two efficacious optimal and adaptive controllers are designed to compensate the undesirable hyperchaotic behaviours. It turns out that fractional calculus techniques leads to more realistic and flexible models with memory effects, which could help us to design more efficient controllers.

## Results and discussion

### The second wave

Since the completion of our study of the first wave and the design of a model explaining the spread of pandemics, the number of infected cases began to rise again. It seems we are almost starting from scratch. This time, a stronger outbreak of the pandemic hit Egypt as from the beginning of the fall. In fact, worldwide epidemiologists when witnessing the first wave suggested the possibility of a much heavier reoccurrence. In addition to the oncoming winter, one of their arguments is based on historical evidence like the second outburst of the Spanish flu with a lethal eruption claiming more than 50 million lives. Again, will there be a third wave of COVID-19 like the latter which struck in four successive waves? No one can speculate its happening. All of these questions are worth exploring and the authors felt obliged to investigate this new situation; and whether they should reverse back to their original model for the purpose of revision and rectification. Fortunately, the model proved to be still sound.

Why a second wave occurred is one major question. Among the many reasons is the rapid lifting of the lockdown, also the irresponsible ease of the restrictions on the part of the individual, his relaxing behavior and negligence in taking the necessary precautions; thinking that severity of the disease is over. We started seeing fewer people wearing masks, more gathering indoors to eat, drink, and observe religious practices or celebrate and socialize. The authorities themselves, at one time, didn't seem to stress upon the measures that people should consider for their safety; for taking the matter too seriously has a serious fallback on the economic situation causing massive disruption of jobs and affecting employment issues. Even if some healthcare responsible believe that the government had decided for a premature reopening to avoid a financial crisis of its citizens along with some mild preventive measures, the effects of that change in policy will take a month or more to be seen. Besides, several cycles of infection/recovery must occur before a noticeable increase shows in the data.

In the authors view, the herd immunity is inevitable if no medical intervention is adopted, and the disease will take its due course in several consecutive waves. In fact, being vaccinated does not provide the person with a proper level of protection; neither does it stop him from transmitting the disease. What the country hopes for by vaccination is to flatten the curve avoiding a vast spreading of the pandemic in order to allow the physicians to cope well and not to overburden the healthcare system. For letting the virus circulate freely among the public can result in either hundreds of thousands of deaths or to the least an everlasting damage to the patients' health. This is why scientists are eagerly trying to determine whether people recovering from COVID-19 get a permanent immunity or that it only lasts for a while and they can get the disease again; resulting in even more death and disability ?.

Like the rest of the world, the first wave in Egypt subsided noticeably around the beginning of October making the Egyptians more relaxed so as not to take the simplest precautions anymore. They started taking off their masks being unaware of the severity of the illness. In fact, everybody felt that the virus had been brought under control and that the cases had fallen substantially. But then, a sustained rise in infections started to be quickly noticed and the Egyptians were compelled this time to show more concern. This switching from taking preventive measures to a complete relaxation and then back again to taking the necessary precautions is not an odd behavior in any society. It is called in science *negative feedback mechanism* whereby any physical/social phenomenon in nature oscillates between positive/high and negative/low until it is brought to a standstill/stabilized situation. Whether the virus this time—like it was announced prematurely—is less deadly but spreads more rapidly; or whether it is still lethal is debatable. We see around us that many are dying. It seems that subsequent rounds of pandemics are sometimes worse than the first like in the case of the Spanish flu killing in a matter of months between 50–100 million people. We know well that viruses tend to mutate and spread anew in different strains. It was found [25], using assisted models with different intervention scenarios, that the likelihood of an additional round of illness and death by COVID-19 can increase significantly when relaxing the preventive interventions used to limit the spread of the pandemic and that the virus has the potential to re-emerge and produce second and possibly even third waves of disease. In fact, the virologists do not rule out the possibility that it might even mutate into something more resilient; thus more lethal, before a potent vaccine becomes widespread. In fact, a group of molecular epidemiologist [26] at the University of Bern in Switzerland, identified a prominent variant of the virus which seemed to have originated in Spain by late summer and is reckoned to be the one to have accounted for 60–80% of all second wave cases in the United Kingdom as well as in other countries.

Going back to our original study of the first wave, the model predicted that the total cumulative number of infected cases *N* will reach near saturation an estimated value for $P = 105,000$ by October as the final epidemic size (the actual figure announced then was 104,000 [27]). Moreover, the estimated values given by the model through the course of the spread fit chronologically with the published data, and this is one major advantage in favor of the model.

As we passed this saturation point, the number of infective cases started to rise again driven by the second wave. The latter saturation point serves as a second inflection point upwards in the course of the pandemic; a point at which ${\text{d}}N/{\text{d}}t$ is almost nil; meaning no new noticeable infections (the first inflection point calculated from Eq. (8) was after almost 82 days from the first of April; a point at which ${\text{d}}N/{\text{d}}t$ is maximum). Note that *N* is a cumulative number that never drops; it either increases or remains almost constant for a while.

Our next task is to estimate the values of both *c* and *P* for this second wave. Based on these estimates, one can expect a second peak which will become a third inflection point (at which ${\text{d}}N/{\text{d}}t$ is another local maximum). Consequently a second saturation point will also be expected according to the model which will be a fourth inflection point at which ${\text{d}}N/{\text{d}}t$ has a local minimum.

To calculate the values of *c* and *P*, we resorted to the actual graph of $N(t)$ published in [27] and which facilitated this time our task enormously. The reason is that for the first wave, as we were about to submit our paper, the pandemic was showing that there a peak on its way. Not knowing when, we had no time to do an analysis. This time, the date of the peak has already passed in the first of January. So we decided to consider the period from the first of November until the first of January ($t_{{\text{m}}} = 60$). The reason for taking the first of November as the starting point is that the curve in [27] was seen to begin accelerating upwards quite noticeably announcing a new wave. The total number of reported cases was reaching then 107,736 from the very beginning of the outbreak, whereas the number of active cases was given as 1903 infectives in the first of November. Since the number of the recovered must be minimal then, the latter figure was taken as the value for $N_{0}$. The value at the peak was given from [27] as $N(t_{{\text{m}}} ) = 139,471$. To compare it with the model, this value is adjusted to 139,471–107,736 + 1903 = 33,638. From Eq. (8), since $t_{{\text{m}}} = 60$, $P - N_{{\text{o}}} = N_{{\text{o}}} {\text{e}}^{60c}$. Thus from Eq. (5), $N(t_{{\text{m}}} ) = P/2$; from which we obtain that *P* = 67,276. By adding the datum value gives the final epidemic size *P* = 67,276 + 107,736–1903 = 173,109. The value of *c* becomes equal to 0.059 which is about the same of the first wave. Finally, assuming that the date at which the pandemic spread will start to subside noticeably is when *N* approaches *P*, say $N = 0.9P$. Equation (5) gives 97 days from the first of November; which is about early February. This matches with what we heard then that the spread has noticeably decreased. However, if we consider the second wave to be almost finished when only one person remains uninfected yet out of every thousand of those who will become eventually infected ($N = 0.999P$), then Eq. (5) gives 177 days, i.e. until the end of April 2021.

**Fig. 4:**
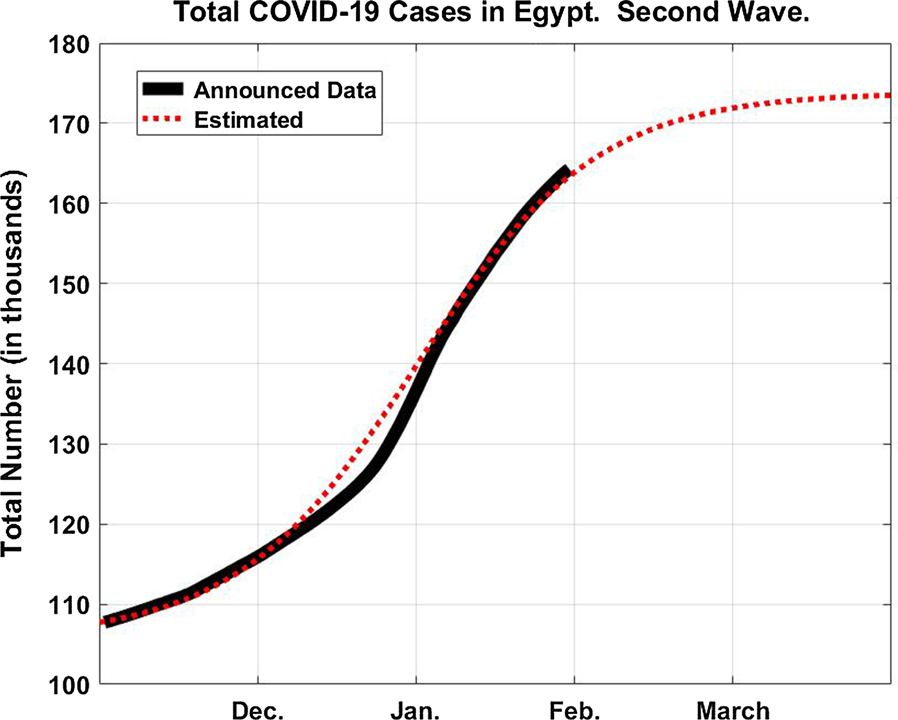
The total number of COVID-19 cases in Egypt in the second wave assuming *P* = 173,000

**Fig. 5:**
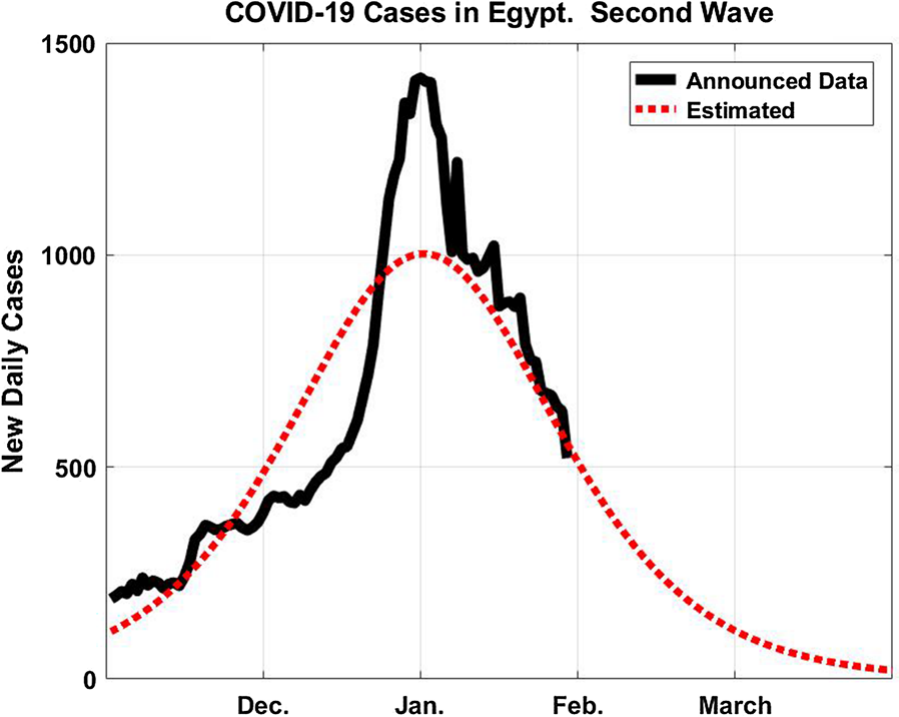
The daily COVID-19 cases in Egypt in the second wave assuming *P* = 173,000

Figure 4 shows the total number of COVID-19 cases in Egypt for the second wave until saturation to be 173,000 while Fig. 5 gives the daily COVID-19 cases starting from the first of November until we intend to submit our revised manuscript.

## Conclusions

In this article, a model has been adopted to predict the spread of the COVID-19 pandemic in Egypt. It is based on some premises that is applicable to many pandemics. It is found that during the sudden outbreak, the number of people who are to contract the disease is expected to rise exponentially. Later it will begin to be curbed automatically after passing a point crucial to the spread. The model was found to fit well with the data published by the Egyptian authorities. A comparison is made with the classical SIR model showing a great similarity between both models. On the contrary, the model we used is advantageous as *P* is a parameter that is allowed to be changed to fit certain acknowledged data like in the statistical models used for short-term forecasts and quick projections. To assess whether the model is superior to other models is not at all a trivial or easy task. However, it is simple and the mathematics involved is straightforward. Besides, the only three issues in our view that are vital to investigate in any model are: how to explain mathematically the graph of *N*(*t*), how to expect the timing of the inflection point as well as the final epidemic size. Again, the model is a long forecasting model in the sense that it can expect the number *N* from the beginning of the outbreak until the pandemic starts to vanish substantially. Yet it is a model valid only for one cycle of the spread, i.e. it cannot forecast a next wave. Like the rest of the models, it follows a Black Box approach and do not consider the mechanism of transmission of a disease. For example, it does not differentiate like many models between direct and indirect mode of transmission. It also supposes that there is a homogeneous mixing of the population irrespective of any age structure. Neither it considers the variability in the transmission/infectivity process or the variation in the incubation period, etc.

Finally, whether we shall witness a third wave or not, it is always possible; nobody can exclude it. Once we reach the fourth inflection point of the second wave at which the daily rate of the infectives becomes minimal that their number may start to rise again predicting a new wave. So, nobody can really tell whether the pandemic will subside eventually and indefinitely or that the world shall witness even multiple reoccurrences. Most likely, the virus came to stay, and it will keep mutating so that the vaccine will turn out to be like the seasonal flu needing to be changed all the time. Although the authors considered the period from the beginning of November until the end of the year, they kept tracing the curves until the end of January. The reader should also note that the figures estimated can change under medical interventions like the vaccination of the population. At last, the authors can recommend the following classical references [28–31] to the reader interested in widening his scope about mathematical modeling of infectious diseases. 

+ +*Herd immunity* is a health term agreed upon by epidemiologists and virologists meaning that when enough people (reckoned to be about 70% of the population) have been immune from a disease by some reason or another (contracting it and forming antibodies, or being vaccinated, etc.…), the community becomes protected from the outbreaks of that disease at least for a while.

## Data Availability

The data used in this study can be found in “COVID-19, estimation updates, published by the University of Washington Institute for Health Metrics and Evaluation, predictions since June 15, 2020.” http://www.healthdata.org/covid/updates. The data is also available in ref [20].

## Abbreviations

COVID-19: Corona virus disease 2019
IHME: Institute for Health Metrics and Evaluation
SIR: Susceptible–infected–recovered
SEIR: Susceptible–exposed–infectious–recovered
SEIRS: Susceptible–exposed–infectious–recovered–susceptible
WHO: World Health Organization
